# *In vitro* activity of ten essential oils against *Sarcoptes scabiei*

**DOI:** 10.1186/s13071-016-1889-3

**Published:** 2016-11-22

**Authors:** Fang Fang, Kerdalidec Candy, Elise Melloul, Charlotte Bernigaud, Ling Chai, Céline Darmon, Rémy Durand, Françoise Botterel, Olivier Chosidow, Arezki Izri, Weiyi Huang, Jacques Guillot

**Affiliations:** 1 Parasitology Department, College of Animal Science and Technology, Guangxi University, Nanning, China; 2 Research group Dynamyc, EA 7380 EnvA, UPEC, UPE, Maisons-Alfort & Créteil, France; 3 Parasitology-Mycology Department, AP-HP, Hôpital Avicenne, Bobigny, France; 4 Dermatology Department, AP-HP, Henri Mondor Hospital, UPEC, Créteil, France; 5 Guangxi Key Laboratory of Traditional Chinese Medicine Quality Standards, Guangxi Institute of Traditional Medical and Pharmaceutical Sciences, Nanning, China

**Keywords:** *Sarcoptes scabiei*, Essential oils, Contact, Fumigation bioassay

## Abstract

**Background:**

The development of alternative approaches in ectoparasite management is currently required. Essential oils have been demonstrated to exhibit fumigant and topical toxicity to a number of arthropods. The aim of the present study was to assess the potential efficacy of ten essential oils against *Sarcoptes scabiei*.

**Methods:**

The major chemical components of the oils were identified by GC-MS analysis. Contact and fumigation bioassays were performed on *Sarcoptes* mites collected from experimentally infected pigs. For contact bioassays, essential oils were diluted with paraffin to get concentrations at 10, 5, and even 1% for the most efficient ones. The mites were inspected under a stereomicroscope 10, 20, 30, 40, 50, 60, 90, 120, 150, and 180min after contact. For fumigation bioassay, a filter paper was treated with 100 μL of the pure essential oil. The mites were inspected under a stereomicroscope for the first 5min, and then every 5min until 1h.

**Results:**

Using contact bioassays, 1% clove and palmarosa oil killed all the mites within 20 and 50min, respectively. The oils efficacy order was: clove > palmarosa > geranium > tea tree > lavender > manuka > bitter orange > eucalyptus > Japanese cedar. In fumigation bioassays, the efficacy order was: tea tree > clove > eucalyptus > lavender > palmarosa > geranium > Japanese cedar > bitter orange > manuka. In both bioassays, cade oil showed no activity.

**Conclusion:**

Essential oils, especially tea tree, clove, palmarosa, and eucalyptus oils, are potential complementary or alternative products to treat *S. scabiei* infections in humans or animals, as well as to control the mites in the environment.

**Electronic supplementary material:**

The online version of this article (doi:10.1186/s13071-016-1889-3) contains supplementary material, which is available to authorized users.

## Background

*Sarcoptes scabiei*, a mite of the family Sarcoptidae, causes a contagious pruritic skin disease in humans and in animals. The human infection is called scabies and is estimated to affect more than 130 million people worldwide at any time [1]. For such, scabies was added to the World Health Organization list of neglected tropical diseases in 2013. The animal infection by *S. scabiei* is devastating and causes significant morbidity and mortality in wild and domestic mammals. It affects more than 100 species worldwide including companion, livestock, and wild animals and is considered as an emerging problem in many countries [2, 3].

In humans, the treatment of *S. scabiei* infection is hindered by the suboptimal efficacy of the few available therapies [4]. In veterinary medicine, the number of available acaricides is much higher but the control of animal ectoparasites (including *S. scabiei*) infections is progressively undermined by the development of resistance to acaricides [5]. In addition, it has been reported that these chemical acaricides induce mild to severe adverse effects [6–8]. For all these reasons, there is an emerging need to develop alternative approaches in ectoparasite management. One of those approaches employs the bioactive effects of plant-derived products. In a recent review, George et al. [6] described the potential pesticidal and repellent effect of plant-derived products such as essential oils, their use, modes of action, and their potential utilization in the prevention and treatment of human and animal ectoparasitoses. Essential oils, which are extracted from plants through steam distillation, are complex natural mixtures containing one to three major components at fairly high concentrations [7]. They have been demonstrated to exhibit fumigant and topical toxicity to a number of insect and mite pests, as well as to fungi and bacteria [7, 8]. In Australia, a topical treatment of 5% tea tree oil combined with benzyl benzoate is used for the treatment of scabies [9].

The aim of this study was to assess the potential efficacy of 10 chemically-characterized essential oils against *S. scabiei* var. *suis*.

## Methods

### Essential oils

The acaricide activity was evaluated for 10 essential oils (Table 1). Each of them was diluted with paraffin oil to obtain three concentration levels of the tested oil at 10, 5 and 1%. The dilution was performed right before setting for contact bioassays. In another series of tests, pure essential oils were used for fumigation bioassays. The contact and fumigation tests were performed at room temperature (20 ± 3 °C) and at 65 ± 5% of relative humidity, and were replicated three times.

**Table 1:** The ten essential oils evaluated in the present study

Family	Common name	Scientific name
*Lamiaceae*	Lavender	*Lavandula angustifolia*
*Rutaceae*	Bitter orange	*Citrus aurantium amara*
*Geraniaceae*	Geranium	*Pelargonium asperum*
*Myrtaceae*	Tea tree	*Melaleuca alternifolia*
	Clove	*Syzygium aromaticum*
	Eucalyptus	*Eucalyptus radiata*
	Manuka	*Leptospermum scoparium*
*Cupressaceae*	Cade	*Juniperus oxycedrus*
	Japanese cedar	*Cryptomeria japonica*
*Poaceae*	Palmarosa	*Cymbopogon martini*

The main components of the essential oils were analyzed by gas chromatography/mass spectrometry (GC/MS) with an Agilent 5977A Series GC/MSD System. The ability of the columns to separate compounds depends on their stationary phases. According to this principle and considering the potential composition of the oils, a DB-1 MS column (30 m × 0.25 mm × 0.25 μm) was used to analyze lavender, tea tree, geranium, manuka, and bitter orange oils, whereas an HP-INNOWax column (30 m × 0.25 mm × 0.25 μm) was used to analyze clove, eucalyptus, cade, palmarosa, and Japanese cedar oils. Helium was the carrier gas. The chemical structure of each component was identified by comparing its fragmentation patterns seen in mass spectra with standard library data. The relative percentage of the oil components was calculated from the GC peak areas.

### *Sarcoptes* mites

*Sarcoptes* mites were collected from pigs maintained at CRBM (Centre de Recherche Bio Médicale), Maisons-Alfort, France. Pigs were experimentally infected as described by Mounsey et al. [10].

Inoculation was performed by directly inserting mite-infected skin crusts deep into the ear canals of five-week old female piglets. The cutaneous lesions developed first in the ear then spread to the entire body of the pigs. In the present study, mites were collected 9 and 10 weeks post-experimental infection. Skin crusts of the external ear canal were gently removed and placed in a Petri dish. Mites were picked up with a needle one h after crust collection; giving time to the mites to climb out of the crusts (Additional file 1: Figure S1). Alive mites of all stages (adults, nymphs and larvae) were then placed one by one in a new plastic Petri dish [11].

### Contact bioassays

For each experiment, alive mites of all motile stages (*n* = 20) were placed in a plastic Petri dish (3 cm in diameter). In the first experiment, all the pure essential oils included in the present study were successively tested against mites. The following bioassays were conducted using the essential oils that killed all the mites within 1 h of direct contact. The selected essential oils were diluted with paraffin to get concentrations at 10, 5 and even 1% (for the most efficient ones). In each Petri dish, 1 ml of the diluted solution was added in direct contact with the mites. A control Petri dish was inoculated with 1 ml of paraffin oil. The mites were inspected under a stereomicroscope (Nikon©, SMZ645, Lisses, France) 10, 20, 30, 40, 50, 60, 90, 120, 150 and 180 min after inoculation. Mites were considered dead when no movement was seen even after touching it with a needle, and no gut movement was observed over 2 min [12].

### Fumigation bioassays

In a separate experiment, the vapor phase toxicity of the 10 oils was investigated. For each fumigation bioassay, 10 mites of all motile stages were placed at the bottom of a plastic Petri dish (3 cm in diameter). A covering filter paper was put on the lid of the Petri dish and treated with 100 μl of the pure essential oil. A control Petri dish was treated with paraffin oil. All the Petri dishes (tested oils and control) were closed and turned over. The mites stayed firmly attached to the bottom of the Petri dish (Additional file 2: Figure S2). They were constantly inspected under a stereomicroscope for the first 5 min, and then every 5 min for 1h. Mites death was expressed by the absence of movement in the legs and the gut.

### Statistical analyses

The data were analyzed by Kaplan Meier survival curves and the median lethal times (LT_50_) of scabies mites were calculated using JMP 12.0 software. The statistical differences between data obtained with each essential oil and the control for each experiment (contact test with 10, 5 and 1% oil concentrations, and fumigation test) were measured by Log-rank test expressed by *χ* ^2^ results and *P*-values (degree of freedom (df) = 1). *P*-value of ≤ 0.05 was considered significant.

## Results

**Fig. 1:**
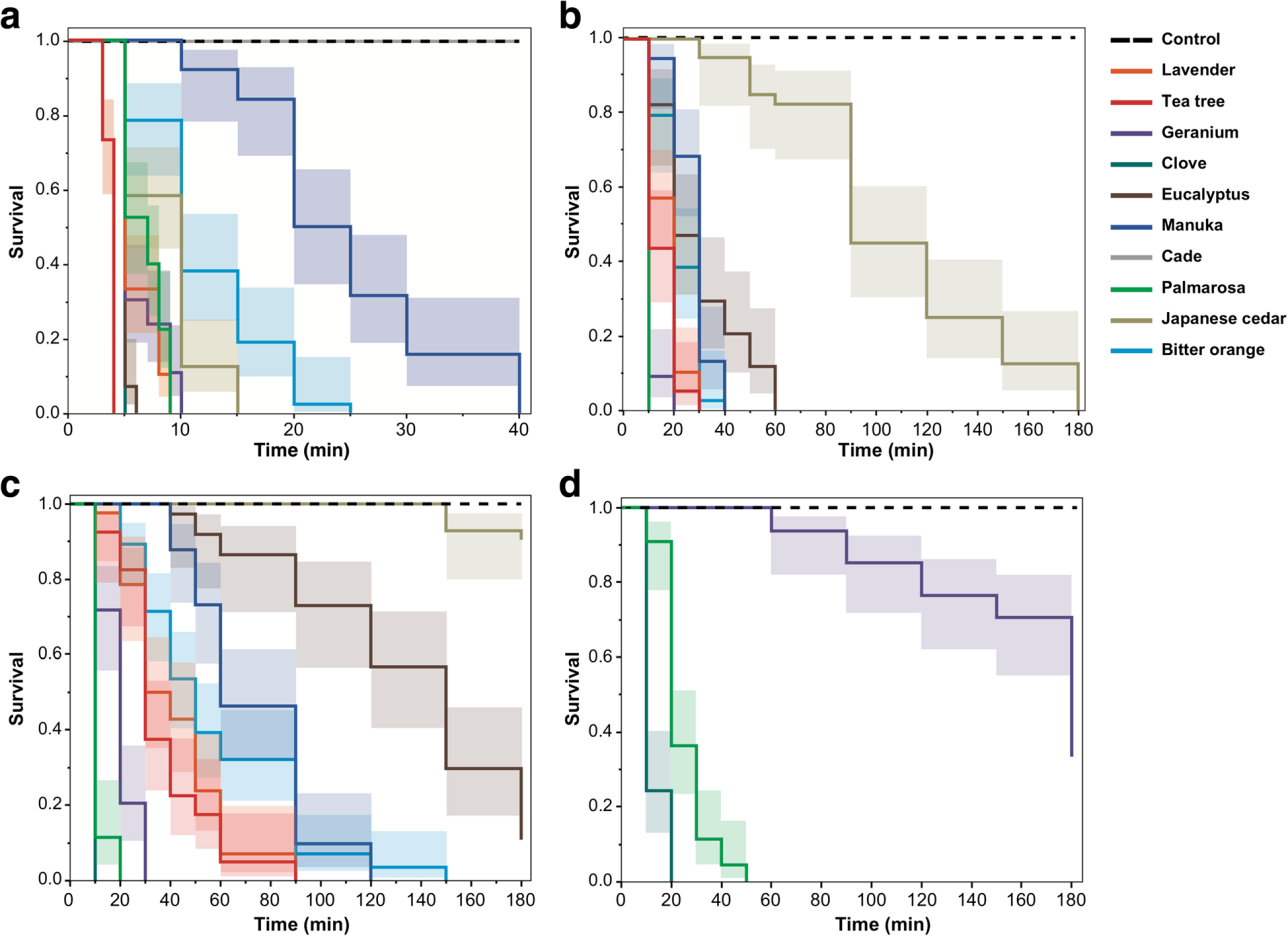
Survival curves of *Sarcoptes scabiei* mites exposed to essential oils. **a** Fumigation test with 10 essential oils. **b** Contact test with 10% of 9 essential oils. **c** Contact test with 5% of 9 essential oils. **d** Contact test with 1% of 3 essential oils. 95% confidence intervals are represented as shaded areas

The major components of the oils analyzed by GC/MS are presented in Table 2. The survival curves of mites exposed to essential oils by direct contact or by fumigation are shown in Fig. 1. The median lethal times are presented in Table 3. In all tests, significant statistical differences were found between each essential oil and the control (*P* < 0.0001) except for the contact test of Japanese cedar oil at 5% concentration (*χ* ^2^ = 3.0741, *P* = 0.0795) (Table 3). Cade oil failed to show any significant effect in both bioassays. Among all the oils tested with the contact bioassay, clove oil demonstrated the best scabicidal effect as its 1% solution killed all mites within 20 min. Based on their median lethal times in contact bioassays (Table 3), the efficacy of these oils can be put in this order: clove > palmarosa > geranium > tea tree > lavender > manuka > bitter orange > eucalyptus > Japanese cedar. No mite died when exposed to pure cade oil for one hour.

**Table 2:** The major components of the ten essential oils tested in the present study

Major components	lvd	bo	grn	tr	cl	ecl	mnk	cd	jc	plm
acetyleugenol					16.3					
cadina-1,4-diene							5.8			
b-cadinene								28.6		
∂-cadinene							6.3			
calamenene							14.0			
l-calamenene								14.4		
caryophyllene								7.5		
citronellol			33.4							
1,8-cineole						78.8				
citronellyl formate			10.9							
o-cymene									5.6	
elemol									17.2	
β-eudesmol									5.1	
eugenol					74.6					
geraniol			12.0							81.4
humulene								5.5		
kaur-16-ene									18.6	
leptospermone							16.3			
linalool	33.0	23.8	5.5							
linalyl acetate	44.1	51.8								
menthone			6.3							
α-pinene									13.9	
sabenene									6.8	
α-terpinene				9.2						
γ-terpinene				15.6						
terpinen-4-ol				37.1					5.9	
α-terpineol		7.0		5.0		8.7				

*Abbreviations*: *lvd* lavande, *bo* bitter orange, *grn* geranium, *tr* tea tree, *cl* clove, *ecl* eucalyptus, *mnk* manuka; *cd* cade, *jc* japanese cedar, *plm* palmarosa

**Table 3:** Median lethal times (LT_50_) of the ten essential oils tested during contact or fumigant bioassays

Essential oil	Contact with a 10% solution			Contact with a 5% solution			Contact with a 1% solution			Fumigation		
	LT_50_ ± SD	*χ* ^2^	*P*	LT_50_ ± SD	*χ* ^2^	*P*	LT_50_ ± SD	*χ* ^2^	*P*	LT_50_ ± SD	*χ* ^2^	*P*
Lavender	20.0 ± 6.6	94.7	<0.0001	35.0 ± 20.0	95.0	<0.0001	–	–	–	5.0 ± 1.6	100.7	<0.0001
Tea tree	10.0 ± 6.0	89.0	<0.0001	30.0 ± 18.0	95.3	<0.0001	–	–	–	4.0 ± 0.4	81.5	<0.0001
Geranium	10.0 ± 2.9	94.2	<0.0001	20.0 ± 7.0	87.7	<0.0001	150.0 ± 40.0	37.1	<0.0001	5.0 ± 1.9	102.1	<0.0001
Clove	10.0	64.0	<0.0001	10.0	79.0	<0.0001	10.0 ± 4.3	87.9	<0.0001	5.0	91.0	<0.0001
Eucalyptus	20.0 ± 16.0	92.2	<0.0001	150.0 ± 44.0	69.2	<0.0001	–	–	–	5.0 ± 0.3	91.0	<0.0001
Manuka	30.0 ± 7.5	86.4	<0.0001	60.0 ± 24.0	89.5	<0.0001	–	–	–	23.0 ± 8.7	91.7	<0.0001
Cade	–	–	–	–	–		–	–	–	>60.0		<0.0001
Palmarosa	10.0	82.0	<0.0001	10.0 ± 3.2	85.4	<0.0001	20.0 ± 9.3	96.7	<0.0001	7.0 ± 1.7	92.2	<0.0001
Japanese cedar	90.0 ± 42.0	93.6	<0.0001	180.0 ± 7.8	3.1	0.0795	–	–	–	10.0 ± 3.4	94.4	<0.0001
Bitter orange	20.0 ± 8.0	86.5	<0.0001	50.0 ± 33.0	99.6	<0.0001	–	–	–	10.0 ± 5.4	94.0	<0.0001

*Abbreviation*: *SD* standard deviation

Concerning the fumigation bioassay, the tea tree oil demonstrated the best efficacy since it killed all the mites in only 4 min, whereas in the contact bioassays, the 10 and 5% diluted solutions of tea tree took 30 and 90 min to kill all the mites, respectively. Based on the median lethal times in fumigation bioassays (Table 3), the efficacy of the oils followed this order: tea tree > clove > eucalyptus > lavender > palmarosa > geranium > Japanese cedar > bitter orange > manuka. Cade oil showed no activity against the mites during fumigation bioassays.

## Discussion

Many publications already reported the efficacy of essential oils against a wide range of ectoparasites [13]. In a study on poultry red mites, 56 essential oils were tested by either filter-paper contact bioassay or fumigation bioassay. In the first series, cade and horseradish oils resulted in 100% mite mortality at a concentration of 0.04 mg.cm^-2^ after 24h, whereas with the fumigation bioassays the same result was obtained with cade, clove bud, coriander, horseradish, and mustard oils at 0.28 mg.cm^-2^ after 24 h [14]. In other studies, on *Psoroptes* mites, the *Laurus novocanariensis* oil at 5% concentration killed all the mites after 24 h of contact [15], and 0.5 g/ml and 1 g/ml extracts of *Eupatorium adenophorum* resulted in a LT_50_ of 0.93 h and 1.29 h, respectively [16].

To our knowledge, the research work covering the efficacy of essential oils against *S. scabiei* is limited in number as only eight botanical extracts including essential oils and their components have been tested in vitro. Among them, neem (*Azadirachta indica*) and tea tree (*Melaleuca alternifolia*) oils were specifically examined for the purpose [17–23]. Other natural products of concern include clove (*Syzygium aromaticum*), nugmeg (*Myristica fragrans*), and ylangylang (*Cananga odorata*) oils, as well as plant extracts from *Eupatorium adenophorum*, *Ailanthus altissima* and *Ligularia virgaurea* [12, 16, 24–26]. Although the efficacies of tea tree and clove oils against *Sarcoptes* mites have already been reported, the current study allowed us, for the first time, to compare these two oils together and with other oils. Pasay et al. reported that 1.56% clove oil killed all *S. scabiei* mites after 15 min in contact bioassay [12], which is consistent with the result of the present study (1% clove oil killed all the mites in 20 min). In another study, clove oil also showed toxicity against house dust mite *Dermatophagoides pteronyssinus* [27]. The lethal time of 5% tea tree oil was reported 180 min [18], whereas in our investigation, all mites died after 90 and 30 min of exposure to 5 and 10% tea tree oil, respectively. Furthermore, our findings showed no scabicidal effect with cade oil which was previously demonstrated effective against head lice and poultry red mites [14, 28]. This inconsistency can be attributed to the varied chemical components of the studied essential oils which are influenced by the method of extraction, type of solvent, date of harvesting, as well as the geographical origin and the selected parts of a single plant species [29].

The fumigant effect of essential oils was studied on stored products pests, head lice, ticks, and *Psoroptes* mites [28, 30–32], but never on *S. scabiei*. A notable finding to report here is the strong fumigant efficacy the tested oils have on scabies mites. However, the fumigant efficacy order of the oils did not exactly match their contact efficacy order. Similar discrepancies were reported in studies on contact and fumigant effects of essential oils against agricultural pests [33, 34]. For instance, in contact bioassays, the median lethal times for 1% clove and palmarosa were 10 ± 4.3 min and 20 ± 9.3 min, respectively (Table 3). In fumigation bioassays, tea tree oil (LT_50_ = 4 ± 0.4) was the most effective, followed by clove (LT_50_ = 5) and eucalyptus oils (LT_50_ = 5 ± 0.3) (Table 3). These results suggest that eugenol, a major component of clove oil (Table 2), exhibits strong contact and fumigant effects against *S. scabiei* mites. Geraniol, which is the main component of palmarosa oil, seems to have strong contact effect but much less fumigant effect. On the contrary, terpinen-4-ol, the main component of tea tree oil, and 1,8-cineole, the main component of eucalyptus oil, were much more active by fumigation than by contact. The volatiles of essential oils are mostly terpenoid substances, particularly monoterpenes (C10) [35], and depending on the type of the bioassay method used in the study, the insecticidal or acaricidal effects of monoterpenes could vary. For example, 1,8-cineole, which is a major component of eucalyptus, rosemary, and tea tree oils, showed a toxic effect in fumigation bioassays against the *Sitophilus oryzae* and *Tribolium castaneum* insects, but was much less effective in contact bioassays. Exactly opposite geraniol which had strong contact toxicity but low fumigant activity [34]. Some monoterpenes could play an important role in ectoparasite management thanks to their fumigant efficacy. Linalool, terpinen-4-ol, geraniol, and eugenol possess potent activity against *Psoroptes cuniculi*, whereas linalyl acetate and estragole were almost ineffective against this parasite [31]. Since linalool and linalyl acetate are two main components in both lavender and bitter orange oils (Table 2), their variable efficacy against *S. scabiei*, as demonstrated in the present study, could be attributed to the variation in their percentages in these oils. On the other hand, the discrepancies between contact and fumigation bioassays can also be explained by the mode of absorption of the active ingredient by the mites. In contact bioassays, the toxic compounds directly penetrate the cuticle layer of the arthropod. In fumigation bioassays, the toxic compounds are inhaled *via* the respiratory system. Therefore, compounds which are lipophilic and viscous might have better performance in contact bioassays, whereas in fumigation bioassays, the vapor pressure may determine the compound efficacy [36].

In this study, the observation time was relatively short (180 min for contact bioassays and 60 min for fumigation bioassays). This is the reason why we believe that using a mixture of instars had a limited effect on our results, although females and nymphs were found surviving longer than males and larvae in the environment [11]. Twenty mites were used for each contact bioassay and only 10 mites for each fumigation bioassay. These numbers can be considered low but the mites were observed every 5 or 10 min (for contact or fumigation bioassays, respectively), so including a larger number of mites might have increased the risk of miscount.

New research work has been directed to study the essential oils mechanism of action on *Sarcoptes* mites. For instance, Hu et al. [37] have recently found that 1,8-cineole increases the activities of superoxide dismutase and glutathione-s-transferase enzymes, which are involved in the protection mechanism of *S. scabiei* mites. The same compound was also shown to affect the nervous system of the mite by increasing the activity of monoamine oxidase and inhibiting acetylcholinesterase.

Essential oils have extensively been used as fragrances, aromatherapy and in pharmaceutical industry for centuries [29]. Given the historically widespread use of them, we might expect these compounds to be relatively safe. Previous studies showed that the great majority of essential oils is ‘slightly toxic’ or ‘non-toxic’ to mammals [38]. However, adverse skin reactions to essential oils have been documented. A review on patch tests using tea tree oil demonstrated that 1.8% of the participants had positive allergic reactions [39]. For such, preventive measures include lowering the concentration and eliminating the main allergens from the original oils [38].

## Conclusion

The present study demonstrated that several essential oils (except cade oil) have both contact and fumigant effects against *S. scabiei* var. *suis*. Essential oils, especially tea tree, clove (for both fumigant and contact use), eucalyptus (for fumigant use), and palmarosa (for contact use), should be considered as complementary or alternative therapies to chemical acaricides in the treatment of *S. scabiei* infections, as well as in the environmental control of the mites.

## Additional files

Additional file 1: Figure S1. Thousands of mites on the crusts collected from the ear canal of the pig model. (JPG 639 kb)
Additional file 2: Figure S2. The mites in fumigation bioassay observed under a stereomicroscope. The mites stayed firmly attached to the bottom of the Petri dish which has been turned over. (JPG 400 kb)

## Abbreviations

CRBM: Centre de Recherche BioMédicale
LT: Lethal time
