# Supplementary figures and images for: In vitro activity of ten essential oils against Sarcoptes scabiei
Source: Parasit Vectors. 2016 Nov 22;9:594. doi: 10.1186/s13071-016-1889-3 (PMC5120413; doi:10.1186/s13071-016-1889-3)

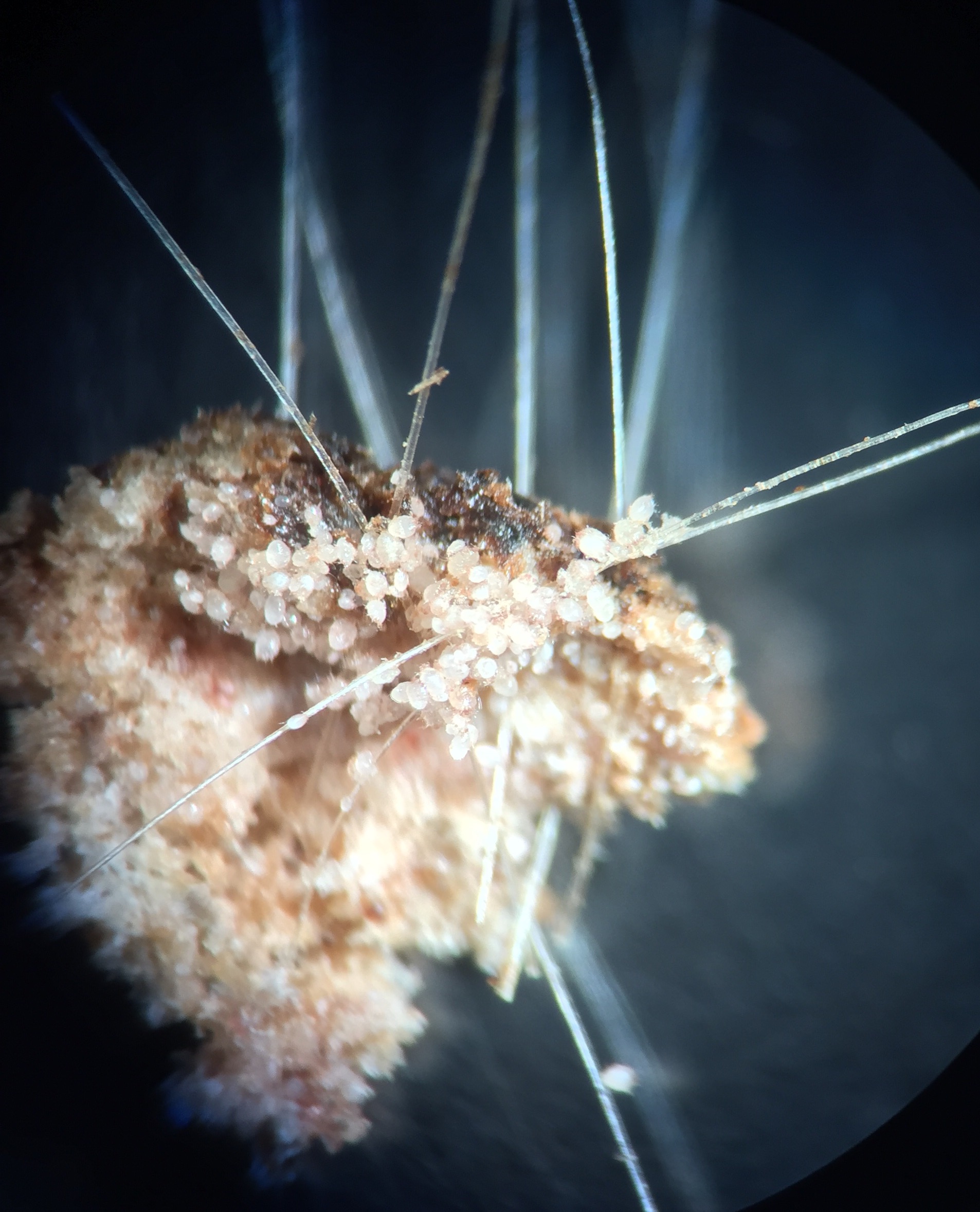

Supplement: Additional file 1: Figure S1. — Thousands of mites on the crusts collected from the ear canal of the pig model. (JPG 639 kb) [file 13071_2016_1889_MOESM1_ESM.jpg]

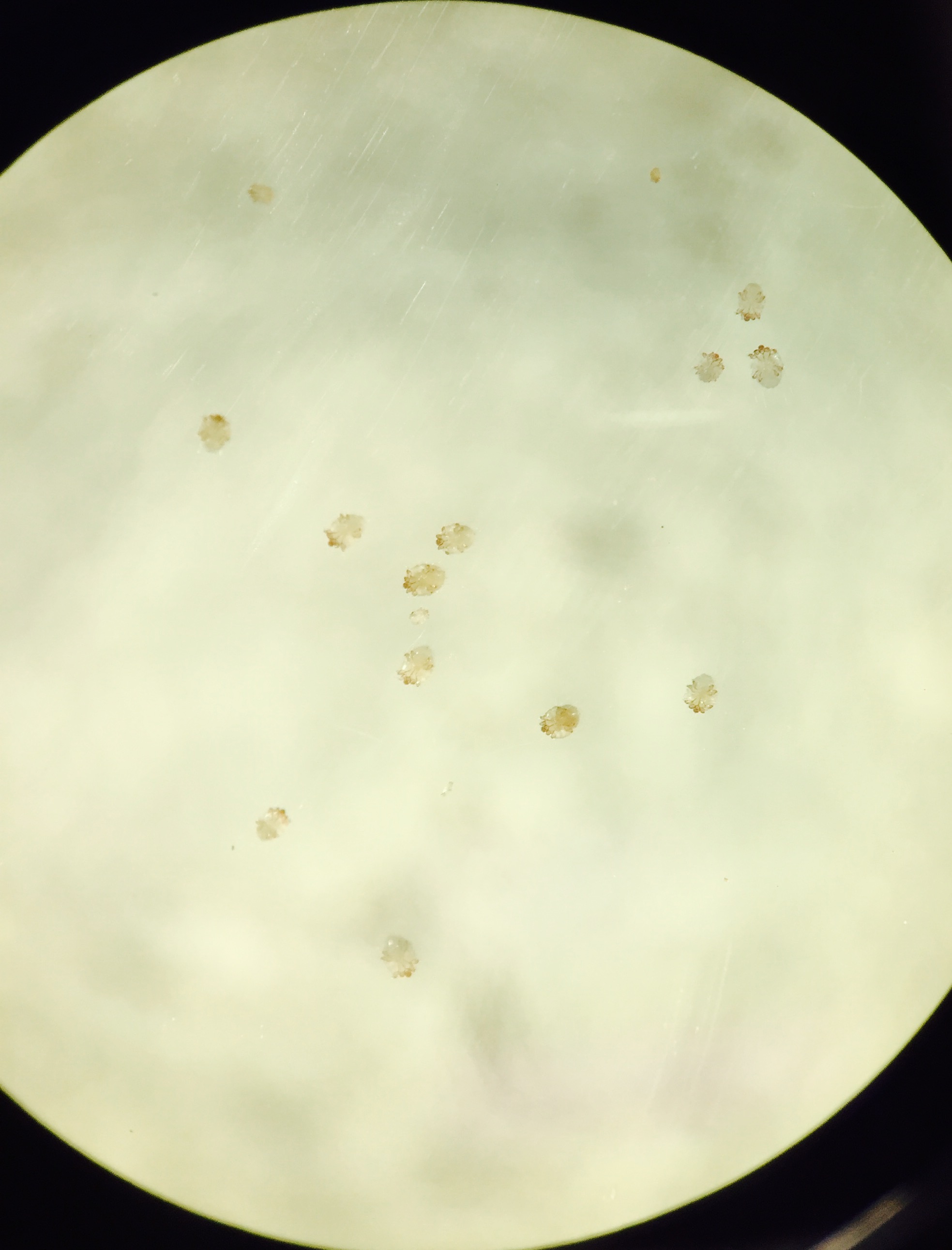

Supplement: Additional file 2: Figure S2. — The mites in fumigation bioassay observed under a stereomicroscope. The mites stayed firmly attached to the bottom of the Petri dish which has been turned over. (JPG 400 kb) [file 13071_2016_1889_MOESM2_ESM.jpg]
